# Supplementary material for: Impact of vaccinations, boosters and lockdowns on COVID-19 waves in French Polynesia
Source: Nat Commun. 2023 Nov 13;14:7330. doi: 10.1038/s41467-023-43002-x (PMC10643399; doi:10.1038/s41467-023-43002-x)
Supplement: Supplementary file 3 — Reporting Summary [file 41467_2023_43002_MOESM3_ESM.pdf]

nature portfolio

Corresponding author(s): Lloyd AC Chapman

Last updated by author(s): Oct 13, 2023

Reporting Summary

Nature Portfolio wishes to improve the reproducibility of the work that we publish. This form provides structure for consistency and transparency in reporting. For further information on Nature Portfolio policies, see our Editorial Policies and the Editorial Policy Checklist.

Statistics

For all statistical analyses, confirm that the following items are present in the figure legend, table legend, main text, or Methods section.

n/a	Confirmed	
☐	☒	The exact sample size (*n*) for each experimental group/condition, given as a discrete number and unit of measurement
☒	☐	A statement on whether measurements were taken from distinct samples or whether the same sample was measured repeatedly
☒	☐	The statistical test(s) used AND whether they are one- or two-sided<br>*Only common tests should be described solely by name; describe more complex techniques in the Methods section.*
☒	☐	A description of all covariates tested
☒	☐	A description of any assumptions or corrections, such as tests of normality and adjustment for multiple comparisons
☐	☒	A full description of the statistical parameters including central tendency (e.g. means) or other basic estimates (e.g. regression coefficient) AND variation (e.g. standard deviation) or associated estimates of uncertainty (e.g. confidence intervals)
☒	☐	For null hypothesis testing, the test statistic (e.g. *F*, *t*, *r*) with confidence intervals, effect sizes, degrees of freedom and *P* value noted<br>*Give P values as exact values whenever suitable.*
☐	☒	For Bayesian analysis, information on the choice of priors and Markov chain Monte Carlo settings
☒	☐	For hierarchical and complex designs, identification of the appropriate level for tests and full reporting of outcomes
☒	☐	Estimates of effect sizes (e.g. Cohen's *d*, Pearson's *r*), indicating how they were calculated

Our web collection on statistics for biologists contains articles on many of the points above.

Software and code

Policy information about availability of computer code

Data collection	No data collection was performed in this study, only secondary analysis of already collected COVID-19 surveillance data, and therefore no data collection software was used.
Data analysis	All data were analysed and fitted in R version 4.1.0, using the odin (v1.3.2), dust (v0.11.24), odin.dust (v0.2.16) and mcstate (v0.9.0) R packages. All of the code required to process the data, fit the model, run the counterfactual simulations and visualise the results is publicly available at: https://github.com/LloydChapman/covid_multi_strain.

For manuscripts utilizing custom algorithms or software that are central to the research but not yet described in published literature, software must be made available to editors and reviewers. We strongly encourage code deposition in a community repository (e.g. GitHub). See the Nature Portfolio guidelines for submitting code & software for further information.

Data

Policy information about availability of data

All manuscripts must include a data availability statement. This statement should provide the following information, where applicable:

- Accession codes, unique identifiers, or web links for publicly available datasets
- A description of any restrictions on data availability
- For clinical datasets or third party data, please ensure that the statement adheres to our policy

All data used in this analysis are publicly available at https://github.com/LloydChapman/covid_multi_strain and https://doi.org/10.5281/zenodo.8320333.

Research involving human participants, their data, or biological material

Policy information about studies with human participants or human data. See also policy information about sex, gender (identity/presentation), and sexual orientation and race, ethnicity and racism.

Reporting on sex and gender	Sex-stratification was not included in the COVID-19 transmission model as data on the sex of confirmed COVID-19 cases was not available.
Reporting on race, ethnicity, or other socially relevant groupings	No social groupings were included in the analysis as this data was not collected.
Population characteristics	Age group was included in the data and modelling due to its important influence on risk of severe COVID-19 outcomes. The population was stratified into 10-year age groups up to the age of 70, with a separate group for individuals 70+ years old. Data on numbers of COVID-19 hospitalisations and deaths was aggregated into one age group for individuals <40 years when comparing with the model output due to low numbers of hospitalisations and deaths in young age groups.
Recruitment	N/A - this study only involved secondary data analysis.
Ethics oversight	Secondary data analysis of COVID-19 data collected in French Polynesia was approved by the London School of Hygiene and Tropical Medicine Observational Research Ethics Committee (ref 28129).

Note that full information on the approval of the study protocol must also be provided in the manuscript.

Field-specific reporting

Please select the one below that is the best fit for your research. If you are not sure, read the appropriate sections before making your selection.

☒ Life sciences ☐ Behavioural & social sciences ☐ Ecological, evolutionary & environmental sciences

For a reference copy of the document with all sections, see nature.com/documents/nr-reporting-summary-flat.pdf

Life sciences study design

All studies must disclose on these points even when the disclosure is negative.

Sample size	Sample sizes are not directly relevant to this modelling study as we did not collect primary data. Our analysis synthesizes information from surveillance data on COVID-19 cases, hospitalisations and hospital deaths in the whole population of French Polynesia, and data from serological surveys on samples of the population. The datasets are sufficiently powered to enable the analyses.
Data exclusions	493 reported COVID-19 cases without an age group (out of 74986 total reported cases, i.e. 0.66% of total reported cases) were excluded from the age-stratified case data used to fit the model, and treated as unreported cases, as under-reporting is accounted for in the fitting of the model.
Replication	The code used to process the data, fit the model, run the counterfactual simulations and visualise the results has been provided to enable replication of the results at: https://github.com/LloydChapman/covid_multi_strain.
Randomization	Randomization is not relevant to this study as it does not contain study groups and most of the data is from routine surveillance of the whole population so includes all available participants.
Blinding	Blinding is not relevant to this study as it does not contain study groups for comparison.

Reporting for specific materials, systems and methods

We require information from authors about some types of materials, experimental systems and methods used in many studies. Here, indicate whether each material, system or method listed is relevant to your study. If you are not sure if a list item applies to your research, read the appropriate section before selecting a response.

Materials & experimental systems

n/a	Involved in the study
☒	☐ Antibodies
☒	☐ Eukaryotic cell lines
☒	☐ Palaeontology and archaeology
☒	☐ Animals and other organisms
☒	☐ Clinical data
☒	☐ Dual use research of concern
☒	☐ Plants

Methods

n/a	Involved in the study
☒	☐ ChIP-seq
☒	☐ Flow cytometry
☒	☐ MRI-based neuroimaging

Plants

Seed stocks

Report on the source of all seed stocks or other plant material used. If applicable, state the seed stock centre and catalogue number. If plant specimens were collected from the field, describe the collection location, date and sampling procedures.

Novel plant genotypes

Describe the methods by which all novel plant genotypes were produced. This includes those generated by transgenic approaches, gene editing, chemical/radiation-based mutagenesis and hybridization. For transgenic lines, describe the transformation method, the number of independent lines analyzed and the generation upon which experiments were performed. For gene-edited lines, describe the editor used, the endogenous sequence targeted for editing, the targeting guide RNA sequence (if applicable) and how the editor was applied.

Authentication

Describe any authentication procedures for each seed stock used or novel genotype generated. Describe any experiments used to assess the effect of a mutation and, where applicable, how potential secondary effects (e.g. second site T-DNA insertions, mosiacism, off-target gene editing) were examined.
